# White matter changes in microstructure associated with a maladaptive response to stress in rats

**DOI:** 10.1038/tp.2016.283

**Published:** 2017-01-24

**Authors:** R Magalhães, J Bourgin, F Boumezbeur, P Marques, M Bottlaender, C Poupon, B Djemaï, E Duchesnay, S Mériaux, N Sousa, T M Jay, A Cachia

**Affiliations:** 1 Physiopathologie des Maladies Psychiatriques, UMR_S 894 Inserm, Centre de Psychiatrie et Neurosciences, Paris, France; 2 Life and Health Sciences Research Institute (ICVS), School of Medicine, University of Minho, Campus de Gualtar, Braga, Portugal; 3 ICVS/3B’s—PT Government Associate Laboratory, Braga/Guimarães, Portugal; 4 Université Paris Descartes, Sorbonne Paris Cité, Paris, France; 5 Faculté de Médecine Paris Descartes, Service Hospitalo Universitaire, Centre Hospitalier Sainte-Anne, Paris, France; 6 Neurospin, I2BM, CEA, Gif/Yvette, France; 7 Laboratoire de Psychologie du développement et de l’Education de l’Enfant, CNRS UMR 8240, Paris, France; 8 Institut Universitaire de France, Paris, France

## Abstract

In today’s society, every individual is subjected to stressful stimuli with different intensities and duration. This exposure can be a key trigger in several mental illnesses greatly affecting one’s quality of life. Yet not all subjects respond equally to the same stimulus and some are able to better adapt to them delaying the onset of its negative consequences. The neural specificities of this adaptation can be essential to understand the true dynamics of stress as well as to design new approaches to reduce its consequences. In the current work, we employed *ex vivo* high field diffusion magnetic resonance imaging (MRI) to uncover the differences in white matter properties in the entire brain between Fisher 344 (F344) and Sprague–Dawley (SD) rats, known to present different responses to stress, and to examine the effects of a 2-week repeated inescapable stress paradigm. We applied a tract-based spatial statistics (TBSS) analysis approach to a total of 25 animals. After exposure to stress, SD rats were found to have lower values of corticosterone when compared with F344 rats. Overall, stress was found to lead to an overall increase in fractional anisotropy (FA), on top of a reduction in mean and radial diffusivity (MD and RD) in several white matter bundles of the brain. No effect of strain on the white matter diffusion properties was observed. The strain-by-stress interaction revealed an effect on SD rats in MD, RD and axial diffusivity (AD), with lower diffusion metric levels on stressed animals. These effects were localized on the left side of the brain on the external capsule, corpus callosum, deep cerebral white matter, anterior commissure, endopiriform nucleus, dorsal hippocampus and amygdala fibers. The results possibly reveal an adaptation of the SD strain to the stressful stimuli through synaptic and structural plasticity processes, possibly reflecting learning processes.

## Introduction

Stress is a major risk factor to the development of severe mental illnesses, including major depression, anxiety,^1, 2^ bipolar disorders and schizophrenia (for review, see Walker *et al.*^3^) and overall one of the more common factors in eliciting dynamic changes in brain states.^4^ Stress is known to trigger the activation of the hypothalamus–pituitary–adrenal axis, culminating in the production of glucocorticoids by the adrenals^5, 6^ that will in turn generate, depending on the individual and the stress stimulus characteristics, adaptive or maladaptive psychoneuroendocrine responses to the stressful stimulus.^7^ In patients with major depressive disorder, dysregulation of the hypothalamus–pituitary–adrenal axis elicits specific and long-lasting functional and structural changes on a network of regions encompassing the hippocampus,^8, 9, 10^ the medial prefrontal cortex^11, 12^ and amygdala.^13, 14^ Subjects with ultrahigh risk for psychosis are particularly sensitive to social stress, life events and daily hassles, which have the potential to trigger psychiatric symptoms; they have an increased basal cortisol level^15, 16^ and a smaller hippocampal volume.^17, 18^ Moreover, stressful life events in non-psychiatric subjects are associated with a gray matter volume decrease in a network encompassing the anterior cingulate cortex, the hippocampus and the parahippocampal gyrus that was observed within a 3-month period.^19^

Animal models have confirmed the drastic effects that stress can have on the brain, including changes in dendritic trees, synaptic plasticity inhibition in the hippocampus and the hippocampal-to-prefrontal pathway,^12, 20, 21^ decreased neurogenesis in the hippocampus^22^ and apoptosis, involving corticosteroids and glutamate receptors.^23^ Taken together, these findings support the effect of stress on structural changes within networks of spatially distributed gray matter regions.

In addition to these regional changes, increasing evidence suggests that stress may also disrupt the structural and functional connectivity within neural networks.^24, 25, 26, 27, 28^ Diffusion magnetic resonance imaging (dMRI) is an advanced technique for examining white matter (WM) anatomy providing insights on the pathway microstructure within neural networks.^29^ A commonly used feature in dMRI studies is fractional anisotropy (FA), which estimates the degree to which tissue organization limits diffusion of water molecules in brain WM.^30^ In animals, different recent dMRI studies investigated changes in diffusion signal associated to chronic stress exposure. Delgado y Palacios *et al.*^31^ were the first to report the effects of stress using *in vivo* dMRI in rats: using diffusion kurtosis imaging (DKI), hippocampus microstructure was revealed to be altered in chronically stressed rats, independently of the hedonic state. More recently, the same team evaluated the mean kurtosis in the PFC, caudate–putamen (CPu) and amygdala in anhedonic-like and resilient rats and found a decrease in the CPu in the anhedonic-like.^32^ In addition, using a similar chronic mild stress (CMS) model, Kumar *et al.*^33^ showed increases in axial diffusion (AD) and radial diffusion (RD) specifically in the CPu and the amygdala of stressed rats. Another study using *in vivo* dMRI showed an increase in the mean diffusion (MD) in the lateral ventricles of chronically stressed rats, although no other changes were found.^27^ Finally, using a mice and a social defeat stress paradigm, Anacker *et al.*^28^ have shown correlations between diffusion metrics and social avoidance correlating positively with FA in the hypothalamus and hippocampus.

Here, we used dMRI and the tract-based spatial statistics (TBSS) approach^34^ adapted to brain rat to investigate the WM microstructure on the entire brain. We selected two strains of rats, Fischer 344 (F344) and Sprague–Dawley (SD), known to have differential response to stress,^35, 36^ and compared their WM microstructure, assessed by four complementary dMRI measures (FA, MD, AD and RD), after exposure to repeated inescapable stress. Repeated exposure to the same stressor very often results in habituation, which leads to a decrease in the hypothalamus–pituitary–adrenal axis response.^37^ In contrast to SD rats, F344 rats show virtually no habituation or adaptation of the corticosterone stress response during repeated stress but an exaggerated acute stress-induced corticosterone secretion^35, 36^ and increased anxiety-related behaviors^38^ with increased amygdala volume.^39^ Such a design allowed studying the effect, but also the responsivity, to stress.

## Materials and methods

### Animals

Experiments were performed with male adult SD (*n*=14) and Fisher 344 (F344; *n*=14) rats (Charles River, Saint-Germain-sur-l'Arbresle, France) at 8 weeks’ age (average of 200 g for SD and 180 g for F344). Rats were housed in groups of two animals with *ad libitum* access to food and water and maintained in a temperature-controlled room, with a light/dark cycle of 12/12 h (lights on at 0600 hours). For each strain, rats were randomly assigned to stressed (*N*=14) and non-stressed (*N*=14) groups. Two animals of the SD strain of the control group were killed before the end of the 2 weeks. The protocols have been approved by the *Comité* *d'* *Éthique en Expérimentation Animale du Commissariat à* *l'* *Énergie Atomique et aux Energies Alternatives*—*Direction des Sciences du Vivant Ile de France* (CETEA/CEA/DSV IdF) under protocol ID 12-058. All procedures were conducted in conformity with National (JO 887–848) and European (86/609/EEC) rules for animal experimentation.

### Stress protocol

The behavioral stress protocol has been previously described elsewhere.^20^ Briefly, rats were placed on an elevated and unsteady platform for 30 min. The platform was positioned 1 m above the ground and illuminated with a high-intensity light source (1500 Lux). While on the platform, animals showed urination, defecation, grooming and freezing. This inescapable stress exposure (called a session) was repeated daily during 15 days between 0900 and 1200 hours.^40^ We measured corticosterone levels for all animals in control condition and after the end of the stress session. Animals were randomly chosen to be stressed or not. This protocol was chosen as we previously demonstrated that it causes with a similar sample size a disruption of synaptic plasticity in the hippocampal-to-prefrontal cortex pathway^20^ and changes in regional brain volumes that are associated with an increase in plasma corticosterone levels.^39^

### Corticosterone immunoassay

The plasma level of corticosterone was assessed as a biomarker of stress in all experiments. Blood samples were collected from the tail under quick anesthesia in basal conditions on day 0 (D0) and 10 min after the end of the stress session at different times (acute stress: D1; repeated stress: D15) for the group exposed to stress. Blood samples for the control group were taken at D15. Anesthesia was induced with 5% isoflurane mixed with oxygen, using a calibrated vaporizer maintained at 2% during the sampling. Samples were centrifuged at 1000 *g* for 15 min, and serum stored at −20 °C. Plasma corticosterone was assessed by immunoassay (Corticosterone Immunoassay, Enzo Life Sciences, Villeurbane, France).

### Tissue preparation

Twenty-four hours after the last day of repeated inescapable stress or after daily handling in control animals, rats were anesthetized with sodium pentobarbital (100–150 mg kg^−1^, intraperitoneally (i.p.)), followed by intracardiac perfusion with physiological NaCl solution and 4% cold paraformaldehyde in 0.01 M phosphate-buffered saline (pH=7.4). After perfusion, the brain was harvested maintaining integrity and stored in 4% PFA in phosphate-buffered saline at 4 °C. Before MRI, the brains were washed into phosphate-buffered saline for 24 h to remove the fixation solution and then placed into a custom-built MRI-compatible tube. The tube was filled with Fluorinert, an MRI susceptibility-matching fluid (Sigma-Aldrich, St Louis, MO, USA).

### Acquisition of diffusion MRI data

Diffusion *ex vivo* data with high spatial and angular resolution were acquired to quantify the subtle changes in the WM microstructure within the entire rat brain. The *ex vivo* MRI acquisitions were performed on a 7 T preclinical scanner (PharmaScan, Bruker, Ettlingen, Germany) using a home‐made quadrature birdcage coil (inside diameter=28 mM). Diffusion images were acquired using a Spin-Echo Multi Shot Echo Planar Imaging (repetition time (TR)=26 s, echo time (TE)=29 ms, 90° excitation pulse followed by a 180° refocusing pulse, 4 segments, 4 averages, total time=24 h16 m 03 s). One hundred four interleaved slices with 0.25 mM thickness were acquired, with a matrix size of 106 × 106, a field-of-view of 25.44 × 25.44 mM and an in-plane resolution of 0.24 × 0.24 mM. Following 10 acquisitions with no diffusion sensitization (*b*=0 s mm^−^^2^), diffusion-weighted images were acquired along 200 noncollinear directions (*b*=4000 s mm^−^^2^). The physicist performing the MRI acquisition was blind to the group allocation (stress versus no stress).

### Preprocessing of diffusion MRI

The dMRI images were reconstructed using an in-house script and visually inspected for brain lesions and artefacts, after which two subjects (one F344 and one SD from the control group) were excluded because of the presence of artefacts. All the data were pre-processed using the FMRIB Software Library^41^ (FSL, http://fsl.fmrib.ox.ac.uk/fsl/) v5.0.6 using the following steps: bias field correction using FAST,^42^ correction of the field inhomogeneity, estimated from b0 images, on all volumes; eddy current distortions and movement correction with fsl ‘eddy_correct’ command-line tool (the first volume without diffusion sensitization was chosen as the reference volume for the affine registration); segmentation of the brain signal using BET:^43^ BET was applied to the mean of the images without diffusion sensitization, with the resulting mask being applied to all volumes. The gradient vector directions were rotated for each subject according to the eddy correct output.^44^

**Figure 1:**
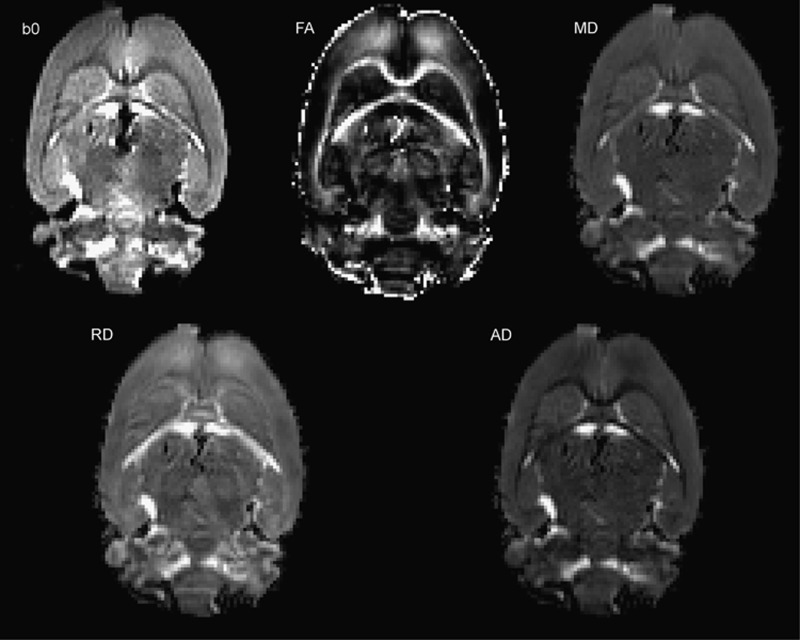
Representative image of the diffusion MRI data (b0 map) and diffusion metrics (FA, MD, RD, AD) in a rat brain. AD, axial diffusivity; FA, fractional anisotropy; MD, mean diffusivity; MRI, magnetic resonance imaging; RD, radial diffusivity.

Tensor fitting and scalar maps were calculated using FSL FDT ‘dtifit’ command line^45, 46^ using the corrected vector directions. These maps were used to obtain the FA, AD, MD and RD maps (see Figure 1). These indexes derived from dMRI provide complementary information of WM microstructure. Although discussed, FA is classically considered to reflect the degree of myelination and axonal density.^47, 48, 49, 50^ AD measures diffusivity parallel to axonal fibers and AD decreases are thought to reflect pathology of the axon itself, such as from trauma or ischemic changes.^47^ RD measures diffusivity perpendicular to axonal fibers and appears to be more strongly correlated with myelin abnormalities, like demyelination, as observed in multiple sclerosis.^51^

### TBSS

Whole-brain voxel-based statistical analysis was performed using the TBSS approach^34^ distributed as part of FSL adapted to the rat brain. The FA maps of all subjects obtained in the tensor-fitting step were aligned into a common space using a study-dedicated template and the nonlinear registration tool FNIRT.^52^ The template was defined as the most representative animal, calculated during the TBSS pipeline as the one that minimizes transformations. Next, all the FA images were averaged and thinned in order to create the mean FA skeleton. A threshold of 0.3 was applied to this skeleton in order to restrict the analysis to the WM tracts, and thus defining the final voxels for analysis. The AD, MD and RD maps of all animals were then warped into this skeleton map using the nonlinear transformations previously calculated for the FA maps.

Statistical analysis for the skeletonized maps of FA, AD, MD and RD was performed using the ‘randomise’ fsl command-line tool, yielding a non-parametric test based on randomization methods. A total of 10 000 random permutations were used with threshold-free cluster enhancement,^53^ and multiple comparison corrections for family-wise error results were considered significant at *P*<0.05. Six different contrasts were calculated, testing for the effect of stress (‘Stress>No stress’ ‘Stress<No stress’), strain (‘SD>F344’ ‘SD<F344’) and stress-by-strain interaction.

**Figure 2:**
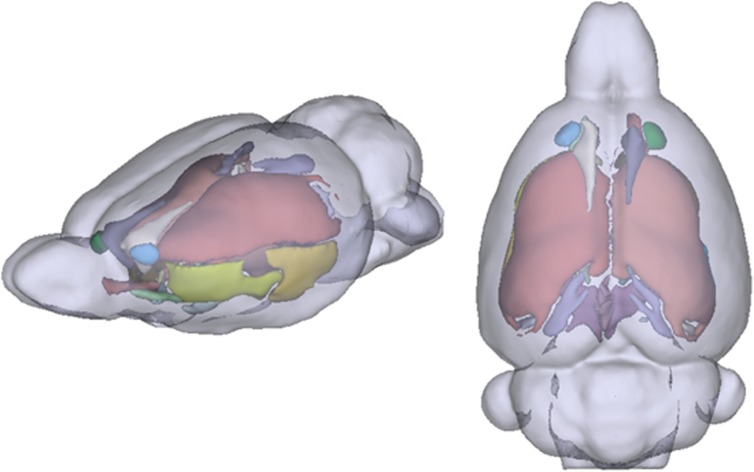
Three-dimensional reconstructions of the white matter skeleton used for TBSS analyses (lateral and top views). White matter tracts were color-coded based on the Paxinos and Watson atlas.^54^ TBSS, tract-based spatial statistics.

Labeling of significant clusters in the FA skeleton was based on the standard Paxinos and Watson atlas^54^ and cross-validated by visual inspection (Figure 2). Descriptive statistics were then calculated separately for each WM bundles.

## Results

### Corticosterone plasma level

**Figure 3:**
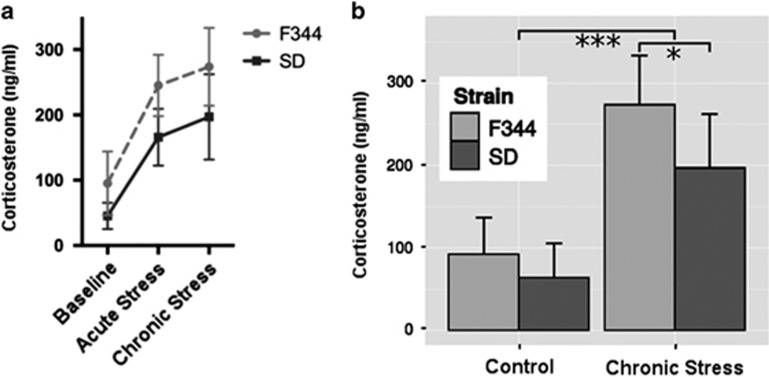
Plasma levels of corticosterone (mean±s.d.) obtained in control and after stress in F344 and SD rats. (**a**) Longitudinal data at baseline (D0), after acute stress (D1) and after chronic stress (D15). (**b**) Comparison between strains before stress (D0) and after chronic stress (D15). Significance levels: *P<0.05, ***P<0.001. F344, Fischer 344; SD, Sprague–Dawley.

As expected, we found a significant main effect of stress on corticosterone plasma levels (F(25, 1)=54.87, *P*=2.7 × 10^−7^) after chronic stress exposure (increase) in both strains ((SD rats: *n*=6, 197±65.34 ng ml^−1^ and F344 rats: *n*=8, 273.75±59.50 ng ml^−1^) when compared with non-stressed rats (SD rats: *n*=6, 63.66±41.86 ng ml^−1^ and F344 rats: *n*=6, 92.50±44.01 ng ml^−1^). There was also a significant difference between the two strains after 15 days of stress exposure with a higher plasma corticosterone level in F344 rats compared with SD rats (*T*(11)=2.18, *P*=0.02; Figure 3). There was no variance difference in corticosterone plasma levels between strains (SD versus F344) in control and stressed animals nor difference between conditions (stress versus no stress) in SD and F344 (Fligner–Killeen non-parametric test of homogeneity of variances, all *P*-values>0.2).

### White matter microstructure

The final number of animals involved in the analysis was as follows: 11 SD rats (four control and seven stressed) and 13 F344 (six control and seven stressed).

The WM skeleton in which statistical tests were conducted was constituted by a total of 6254 voxels. All the statistical tests were done with 23 degrees of freedom.

TBSS analyses revealed no significant main effect of strain (‘F344’ versus ‘SD’) in FA, AD, MD or RD maps (*P*_corrected_>0.05).

**Figure 4:**
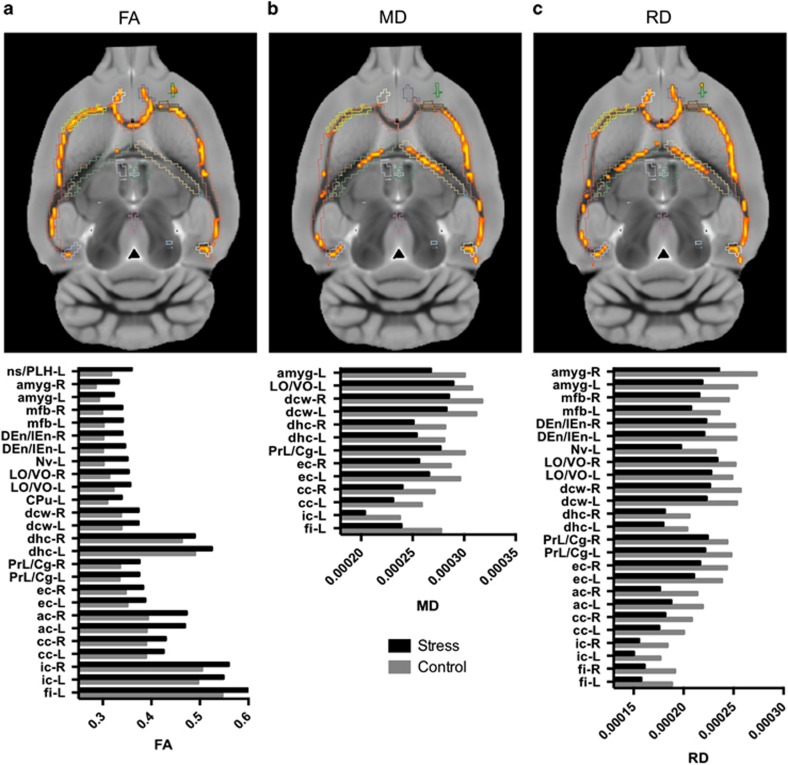
White matter tracts with microstructural differences between control rats and stressed rats. Top panel represents an axial brain slice with voxels with significant main effect of stress (red–yellow scale) superimposed on the white matter skeleton used for TBSS analyses. Bottom panel provides histogram of white matter tracts microstructure (FA, MD or RD) in control (black) and stressed (light gray) rats. Only tracts with significant main effect of stress on FA (**a**), MD (**b**) or RD (**c**) are represented. For illustration purpose, the tracts were slightly dilated. FA, fractional anisotropy; MD, mean diffusivity; RD, radial diffusivity; TBSS, tract-based spatial statistics.

In contrast, we found a main effect of stress (‘control’ versus ‘stress’) in several WM bundles, with increased FA (peak *T*-value=5.720, peak-corrected *P*-value=0.012, cluster size of 3126 voxels) and decreased RD (peak *T*-value=4.621, peak-corrected *P*-value=0.011, cluster size of 3480 voxels) and MD (peak *T*-value=4.598, peak-corrected *P*-value=0.037, cluster size of 1515 voxels) in stressed animals compared with controls (Figure 4). These stress-related differences were distributed over the entire brain and involved WM bundles in posterior and anterior areas, on both hemispheres (Table 1).

**Figure 5:**
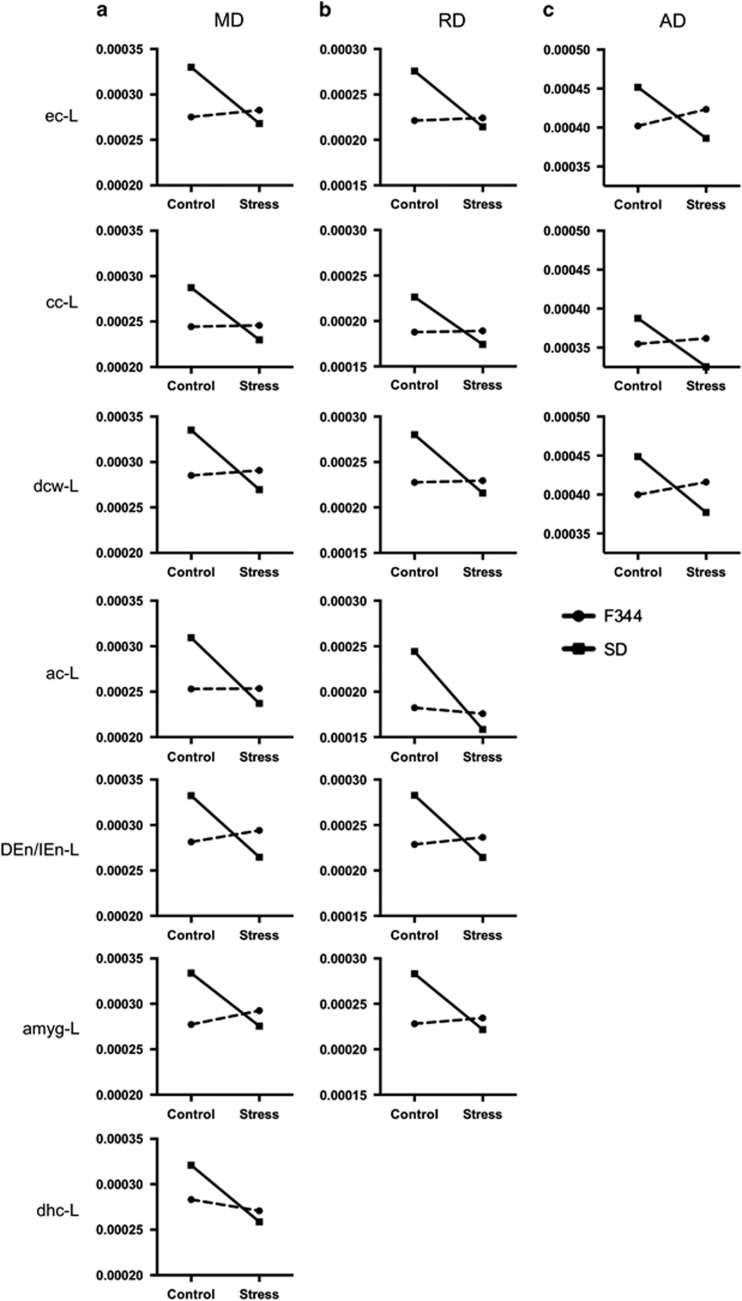
White matter tract changes in microstructure associated with maladaptative response to stress. Interaction graphs provide the mean values of white matter tract microstructure - FA (**a**), MD (**b**) or RD (**c**) - in control (black) and stressed (light gray) animals, in F344 (circles with solid lines) and SD (squares with dotted lines) rats. ac, anterior commissure; amygFib, amygdala fiber; cc, corpus callosum; dcw, deep cerebral white matter; denien, dorsal and intermediate endopiriform nucleus fiber; dhc, dorsal hippocampus commisure; ec, external capsule; FA, fractional anisotropy; F344, Fischer 344; MD, mean diffusivity; RD, radial diffusivity; SD, Sprague–Dawley.

Finally, significant strain-by-stress interactions were found in MD, RD and AD maps (Figure 5), with a stress-related decrease in SD rats and an absence of change in F344 rats. Significant interactions involved WM bundles in the left hemisphere and included the following: the corpus callosum (cc), external capsule (ec) and deep cerebral WM (dcw) for MD, RD and AD measures; the anterior commissure (ac), dorsal and intermediate endopiriform nucleus (DEn/IEn) and amygdala for MD and RD measures; and dorsal hippocampus commissure (dhc) on MD. All statistics related to these results can be found on Table 2.

## Discussion

This diffusion MRI study reveals that 15 days of repeated exposure to the same inescapable stressor in rats leads to microstructural WM changes—increased FA and decreased MD and RD—of several WM bundles distributed in the entire brain. Furthermore, differential stress effects were observed in SD and F344 rat strains, which are known to have a different behavioral and physiological habituation to repeated stress.^35, 36^

Several WM bundles reported in this study (including amygdala fibers, dcw, DEn/IEn fibers, dorsal hippocampus, fimbria of the hippocampus, external capsule and corpus callosum) connect brain areas associated with emotion formation and processing, attention, and learning and memory.^14, 55, 56, 57^ Of note, some of these bundles interconnect the hippocampus (dhc and fi) or connect the hippocampus to the amygdala, prefrontal cortex and anterior thalamic nuclei (ec, cc, StrlCg and dcw), regions consistently reported to be affected by stress.^7, 9, 10, 12, 14^ Changes found in ac, proximal to the olfactory bulb and in Denien^58^ may indicate a stress-related alteration in sensory circuits, possibly because of a readjustment of the perception of their surroundings.

To our knowledge, this is the first study to show the effects of repeated acute stress exposure in two strains with different stress sensitivity and habituation. Indeed, we show decreased MD, RD and AD in several brain bundles in SD rats, whereas no such differences were observed in F344 rats. This is particularly relevant, as SD rats were able to adjust their stress response to the repeated exposure to acute stress (resilience), therefore, showing an adaptive response that may be triggered by the acquisition of coping mechanisms that are paralleled by the decreases in MD, RD and AD, despite the overall increase in FA. In contrast, F344 (nonresilient) rats, which display a maladaptive response, do not reveal significant changes in these parameters. These findings suggest that differential response to repeated acute stressors may be revealed by or are associated with the ability to trigger structural plastic events in WM.

A few preclinical dMRI studies previously reported measurable effects of stress on several brain regions, and in all cases addressing the impact of chronic stress. Indeed, a significant decrease in the mean and radial kurtosis in the hippocampus was detected following CMS in rats.^31^ More recently, the same team reported significant stress-related increases in AD and RD in the CPu and in the amygdala, respectively, along with a mean kurtosis decrease in the CPu in anhedonic-like animals compared with resilient animals.^32^ Such effects were interpreted as the result of axonal degeneration and demyelination within WM bundles with disrupted microstructural spatial coherence. A FA decrease interpreted as a potential loss of myelin sheath was also found in the corpus callosum, bilateral frontal cortex and bilateral hypothalamus in rats after a similar CMS protocol.^33^

Such contrasting results are likely to reflect the temporal dynamics of the stress response (and its successful, or not, adaptation). Yet, we cannot exclude that other methodological differences may also explain the difference in FA change direction, including the stress paradigm (repeated acute stress versus CMS), image acquisition (*in vivo* dMRI versus *ex vivo* data with higher spatial resolution and higher signal-to-noise ratio) or image analysis (measure in *a priori* preselected regions of interest, mostly within gray matter structures versus voxel-wise analysis on the whole WM tracts).

Increased WM FA has been repeatedly associated to learning^59, 60, 61^ via neuronal plasticity processes (for example, synaptogenesis and dendritic branching) and glial remodeling (for example, modification of astrocyte processes).^62^ An increased FA was found in the corpus callosum after a spatial learning task and such increase was supported by significant increases in immune reactivity for a myelin marker, suggesting an increase in the cellular organization and packing of axons or myelin.^59, 63^ More recently, TBSS analysis also revealed higher FA in skilled learning rats in comparison with control^64^ that could be explained by increases in myelination. On the other hand, a reduction in MD was found in both rat hippocampi before and after learning a hippocampal-dependent spatial navigation task.^61^ Data from both human and animal studies indicate the potential for rapid changes in dMRI indices,^61, 65^ suggesting changes in structural plasticity in specific brain regions. The patterns of FA increase/RD decrease are likely related to a tissue density increase due to reshaping of neuronal or glial processes, and/or enhancement of tissue organization, including strengthening of axonal or dendritic backbones and surrounding tissue.^66^ Myelination, known to be modified by experience and maturation,^67, 68^ may also partly explain the RD decrease observed in the stressed rats as RD increases have previously been associated with demyelination processes.^69, 70^ Of note, an activity-dependent myelination has been recently proposed in a human study of motor training, where the FA change in WM was accompanied by adjacent gray matter density alterations.^71^

This study presents some limitations that should be considered. Here the susceptible/resilient differences are achieved by using different strains. We cannot discard the possibility that the mechanisms that lead to different responses to stress within a single strain are different, or if the results found are specific to the SD strain, making its generalization harder. In addition, corticosterone was the only measure used to access the stress response, and although it is known to be one of the more representative markers of stress, the use of complementary behavioral assessment could be beneficial. Other limitations include the lack of direct histological correlations between DTI indices and morphological markers due to the exclusive *ex vivo* approach. *In vivo* longitudinal measurements would have allowed comparisons before and after stress, however, at the expense of signal-to-noise ratio and diffusion MRI spatial and angular resolution.

To conclude, we identified microstructural changes in the key WM tracts like the corpus callosum and the amygdala fibers linked to the frontolimbic circuitry with a functional relevance for cognitive performance and emotional response. Our data demonstrate that SD rats able to adjust to repeated exposure to an acute stress leads to significant changes in dMRI indices. These changes are not well understood, but we demonstrate that dMRI may offer a novel measure of microstructural remodeling occurring in response to stress to further explore the neural basis of adaptive and maladaptive response to stress in rodents and provide quantitative biomarkers to evaluate novel treatments to the protection of stress effects.

## Figures and Tables

**Table 1:** Abbreviations of the white matter tracts investigated in the study

*Abbreviation*	*White matter tract*
ac	Anterior commissure
amygfib	Amygdala fibers
cc	Corpus callosum
dcw	Deep cerebral white matter
denien	Dorsal and intermediate endopiriform nucleus fibers
dhc	Dorsal hippocampus commissure
ec	External capsule
fi	Fimbria of the hippocampus
ic	Internal capsule
inwh	Intermediate white layer
lovo	Lateral orbital cortex/ventral orbital cortex
mfb	Medial forebrain bundle
nsplh	Nigrostriatal bundle/peduncular part of the lateral hypothalamus
nv	Navicular nu basal forebrain
opt	Optic tract
optot	Olivary pretectal nu/nu of the optic tract
prlcg	Prelimbic cortex/cingulate cortex
strfibers	Striatum fibers
strmlfr	Superior thalamic radiation/medial lemniscus/fasciculus retroflexus

White matter tracts were labeled based on the Paxinos and Watson atlas.^54^

**Table 2:** Statistics of the white matter tracts associated with maladaptive response to stress

*White matter tract*	*MD*	*RD*	*AD*
*External capsule (ec)*			
Peak *T*-value	3.62	3.55	3.35
Peak *P*-value	0.041	0.048	0.043
# Voxels	219	26	205

*Corpus callosum (cc)*			
Peak *T*-value	4.17	3.66	3.65
Peak *P*-value	0.041	0.035	0.049
# Voxels	104	114	15

*Deep cerebral white matter (dcw)*			
Peak *T*-value	3.89	3.264	4.39
Peak *P*-value	0.041	0.043	0.048
# Voxels	199	154	117

*Anterior* *commissure* *(ac)*			
Peak *T*-value	4.05	4.41	—
Peak *P*-value	0.043	0.044	—
# Voxels	23	23	—

*Dorsal and intermediate endopiriform nucleus fibers (denien)*			
Peak *T*-value	2.97	2.69	—
Peak *P*-value	0.046	0.048	—
# Voxels	8	7	—

*Amygdala fibers (amyg)*			
Peak *T*-value	2.56	2.50	—
Peak *P*-value	0.042	0.045	—
# Voxels	1	2	—

*Dorsal hippocampus* *commissure* *(dhc)*			
Peak *T*-value	2.72	—	—
Peak *P*-value	0.049	—	—
# Voxels	2	—	—

Abbreviations: AD, axial diffusivity; MD, mean diffusivity; RD, radial diffusivity.

The peak *T*-value, the corresponding peak *P*-value and the number of voxels in the cluster are provided for white matter tracts with significant strain-by-stress interactions on MD, RD and AD.
